# Supplementary material for: Speciation on the Roof of the World: Parallel Fast Evolution of Cryptic Mole Vole Species in the Pamir-Alay—Tien Shan Region
Source: Life (Basel). 2023 Aug 16;13(8):1751. doi: 10.3390/life13081751 (PMC10455883; doi:10.3390/life13081751)
Supplement: Supplementary file 1 [file life-13-01751-s001.zip › Figures S1-S9.pdf]

Supplementary materials

Article

Speciation on the Roof of the World: Parallel Fast Evolution of Cryptic Mole Vole Species in the Pamir-Alay – Tien Shan Region

Aleksey Bogdanov [1], Valentina Tambovtseva [1,*], Sergey Matveevsky [2], and Irina Bakloushinskaya [1,*]

[1] Koltzov Institute of Developmental Biology, Russian Academy of Sciences, 119334 Moscow, Russia; bogdalst@yahoo.com (A.B.); i.bakloushinskaya@idbras.ru (I.B.)

[2] Vavilov Institute of General Genetics, Russian Academy of Sciences, 119991 Moscow, Russia; sergey8585@mail.ru

* Correspondence: tambovceva@idbras.ru (I.B.); i.bakloushinskaya@idbras.ru (V.T.)

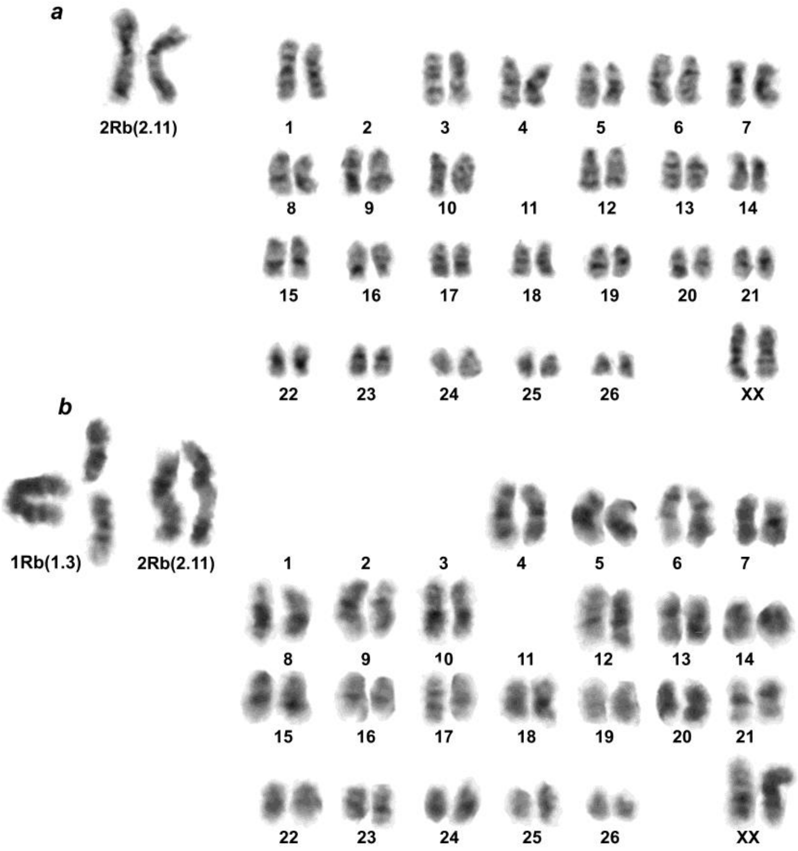

Figure S1. Karyotypes of *E. alaicus* (a) 27522, 2n = 52, #1; (b) 27534, 2n = 51, #4; G-banding.

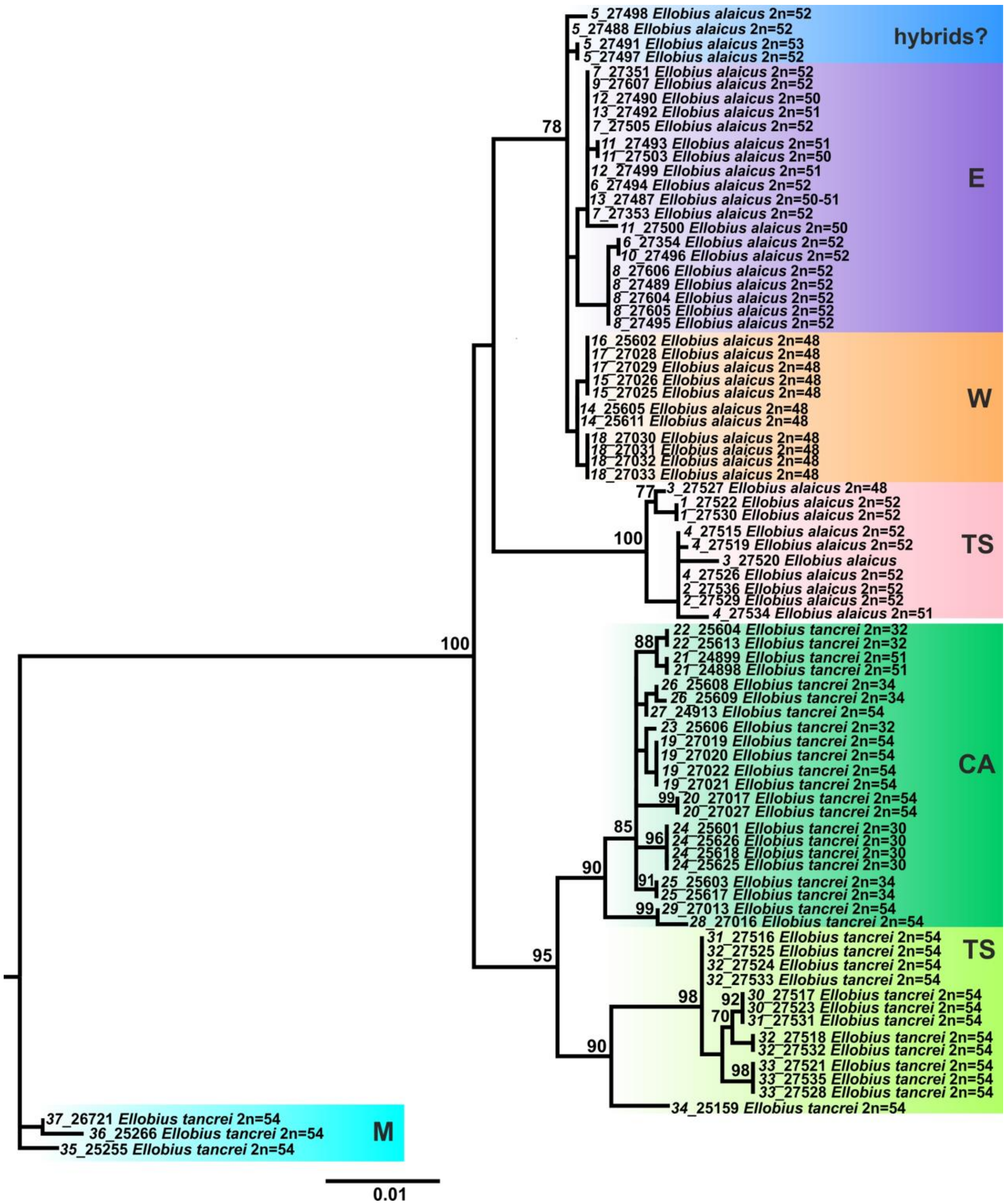

Figure S2. The maximum-likelihood tree based on *cytb* gene sequences of 82 specimens of *E. tancrei* and *E. alaicus*. Samples from *E. tancrei* Mongolian populations were used as an outgroup. Numbers above the nodes correspond to bootstrap support; values <70 are not specified. Sample names are presented as "locality number—individual number". The color selection corresponds to haplowebs: cyan marks Mongolian *E. tancrei* (#35–37); deep green marks *E. tancrei* from Tajikistan (#19-29); bright green marks Tien Shan *E. tancrei* (#30-34); blue marks probable interspecific hybrids from Gulcha vicinities (#5); pink marks Tien Shan *E. alaicus* (#1-4); purple and orange mark "E" (#6-10) and "W" (#14-18) haplotypes of *E. alaicus*, respectively. Populations #11-13 belong to "E" group but demonstrated some features (*IRBP* alleles).

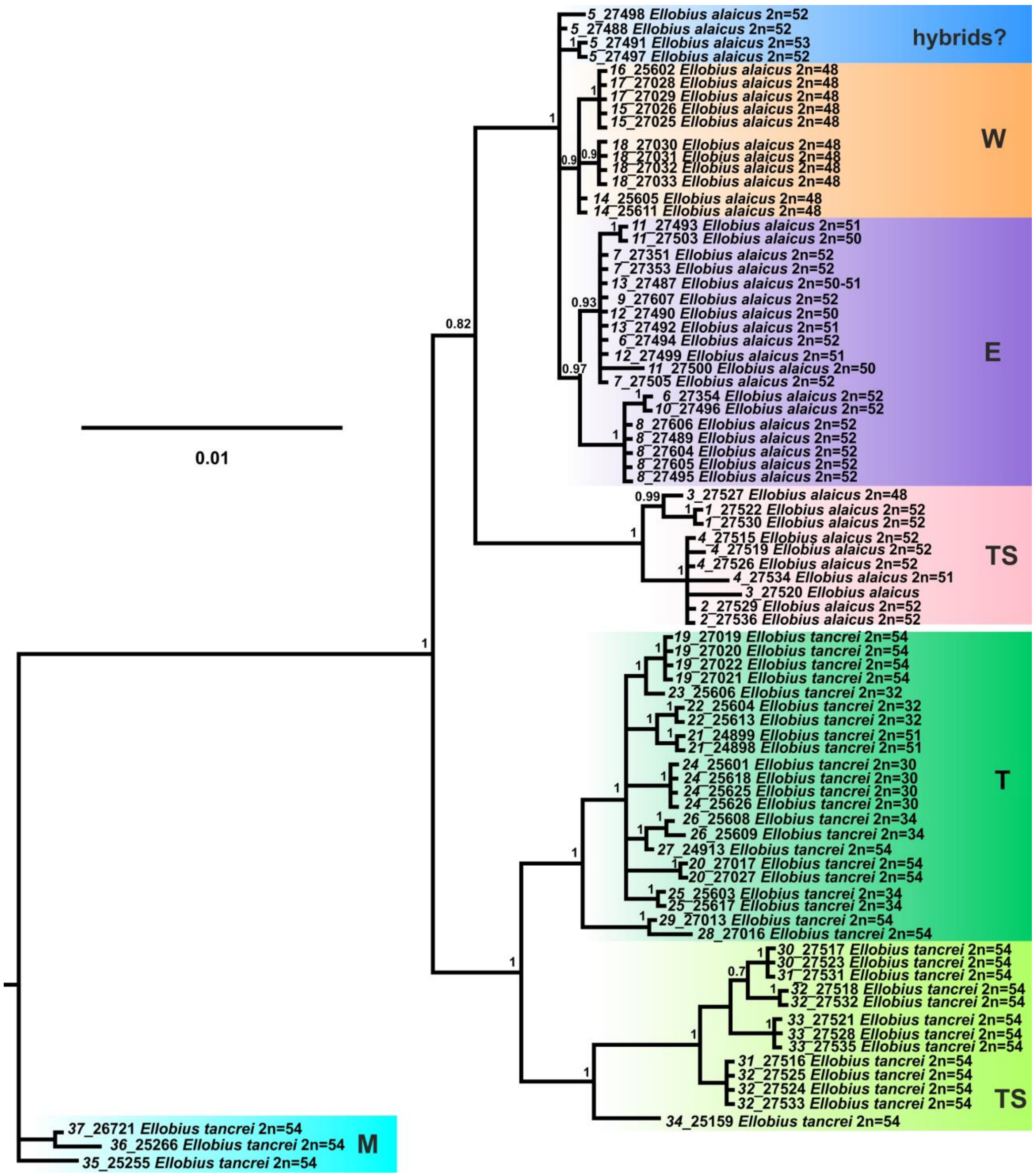

Figure S3. The Bayesian tree based on *cytb* gene sequences of 82 specimens of *E. tancrei* and *E. alaicus*. Samples from *E. tancrei* Mongolian populations were used as an outgroup. Numbers to the right of the nodes correspond to posterior probability values; values <0.70 are not specified. Sample names are presented as "locality number—individual number". The color selection corresponds to haplowebs: cyan marks Mongolian *E. tancrei* (#35–37); deep green marks *E. tancrei* from Tajikistan (#19-29); bright green marks Tien Shan *E. tancrei* (#30-34); blue marks probable interspecific hybrids from Gulcha vicinities (#5); pink marks Tien Shan *E. alaicus* (#1-4); purple and orange mark "E" (#6-10) and "W" (#14-18) haplotypes of *E. alaicus*, respectively. Populations #11-13 belong to "E" group but demonstrated some features (*IRBP* alleles).

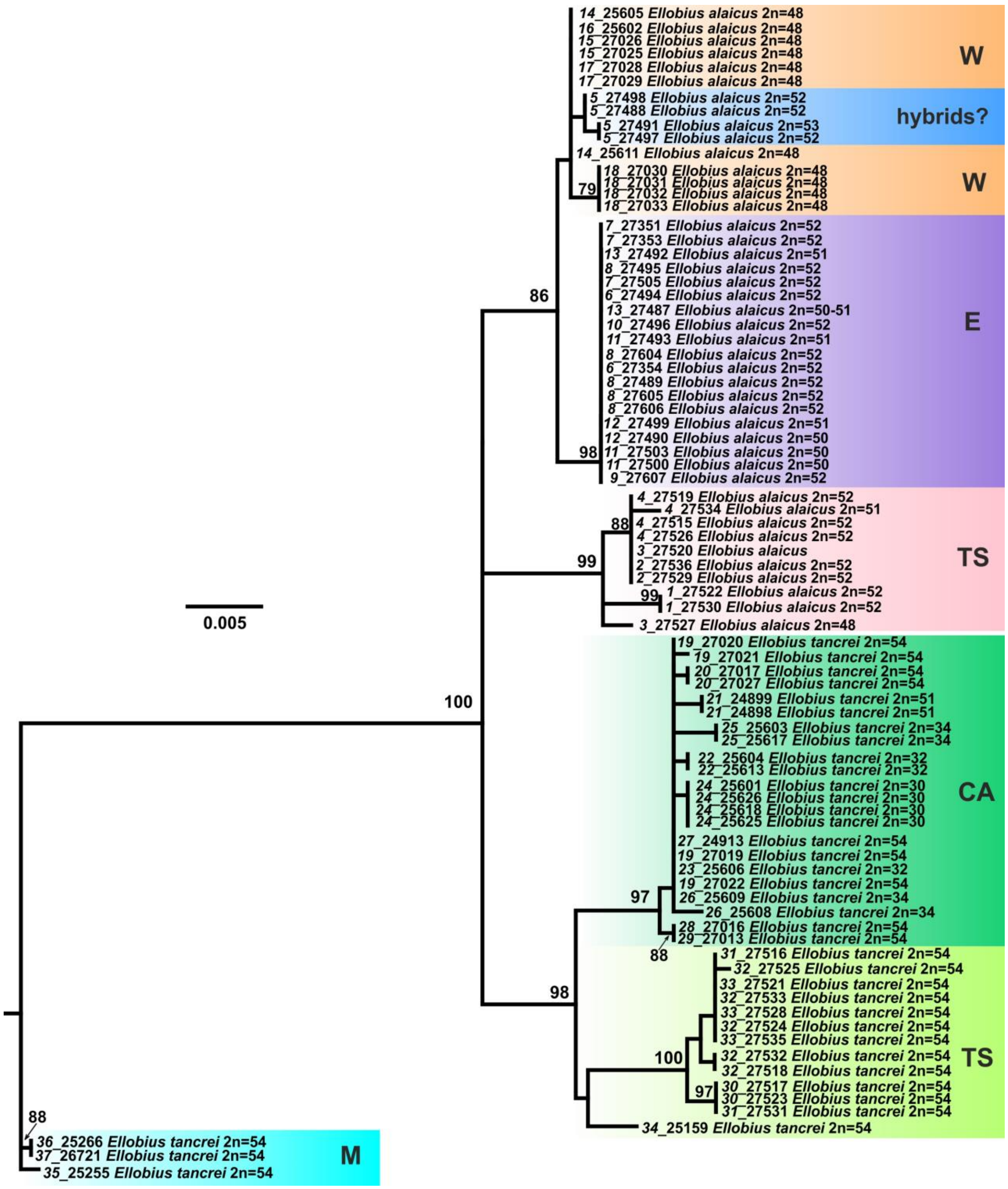

Figure S4. The maximum-likelihood tree based on *COI* gene sequences of 82 specimens of *E. tancrei* and *E. alaicus*. Samples from *E. tancrei* Mongolian populations were used as an outgroup. Numbers above the nodes correspond to bootstrap support; values <70 are not specified. Sample names are presented as “locality number—individual number”. The color selection corresponds to haplowebs: cyan marks Mongolian *E. tancrei* (#35–37); deep green marks *E. tancrei* from Tajikistan (#19-29); bright green marks Tien Shan *E. tancrei* (#30-34); blue marks probable interspecific hybrids from Gulcha vicinities (#5); pink marks Tien Shan *E. alaicus* (#1-4); purple and orange mark “E” (#6-10) and “W” (#14-18) haplotypes of *E. alaicus*, respectively. Populations #11-13 belong to “E” group but demonstrated some features (*IRBP* alleles).

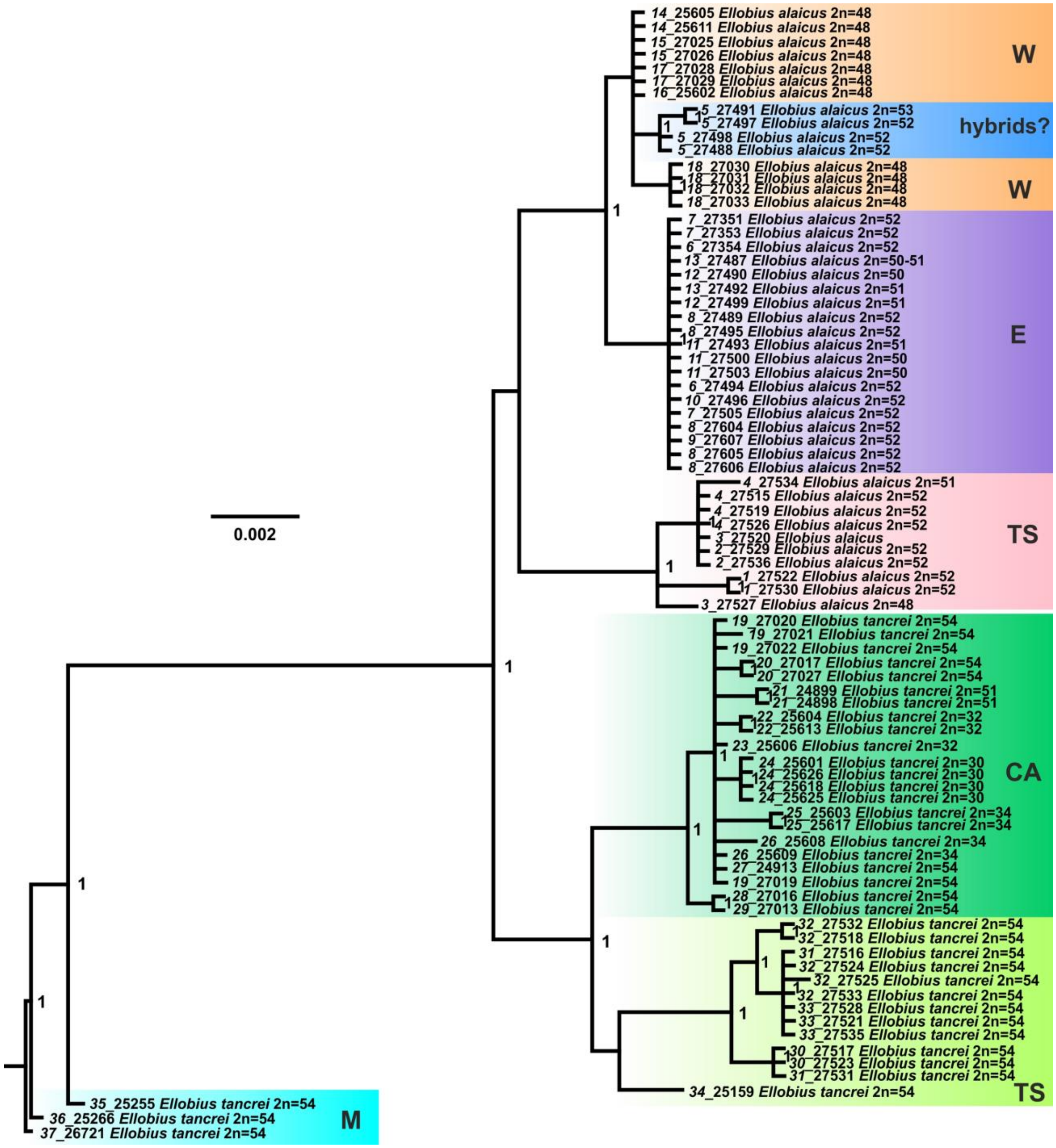

Figure S5. The Bayesian tree based on *COI* gene sequences of 82 specimens of *E. tancrei* and *E. alaicus*. Samples from *E. tancrei* Mongolian populations were used as an outgroup. Numbers to the right of the nodes correspond to posterior probability values; values <0.70 are not specified. Sample names are presented as "locality number—individual number". The color selection corresponds to haplowebs: cyan marks Mongolian *E. tancrei* (#35–37); deep green marks *E. tancrei* from Tajikistan (#19-29); bright green marks Tien Shan *E. tancrei* (#30-34); blue marks probable interspecific hybrids from Gulcha vicinities (#5); pink marks Tien Shan *E. alaicus* (#1-4); purple and orange mark "E" (#6-10) and "W" (#14-18) haplotypes of *E. alaicus*, respectively. Populations #11-13 belong to "E" group but demonstrated some features (*IRBP* alleles).

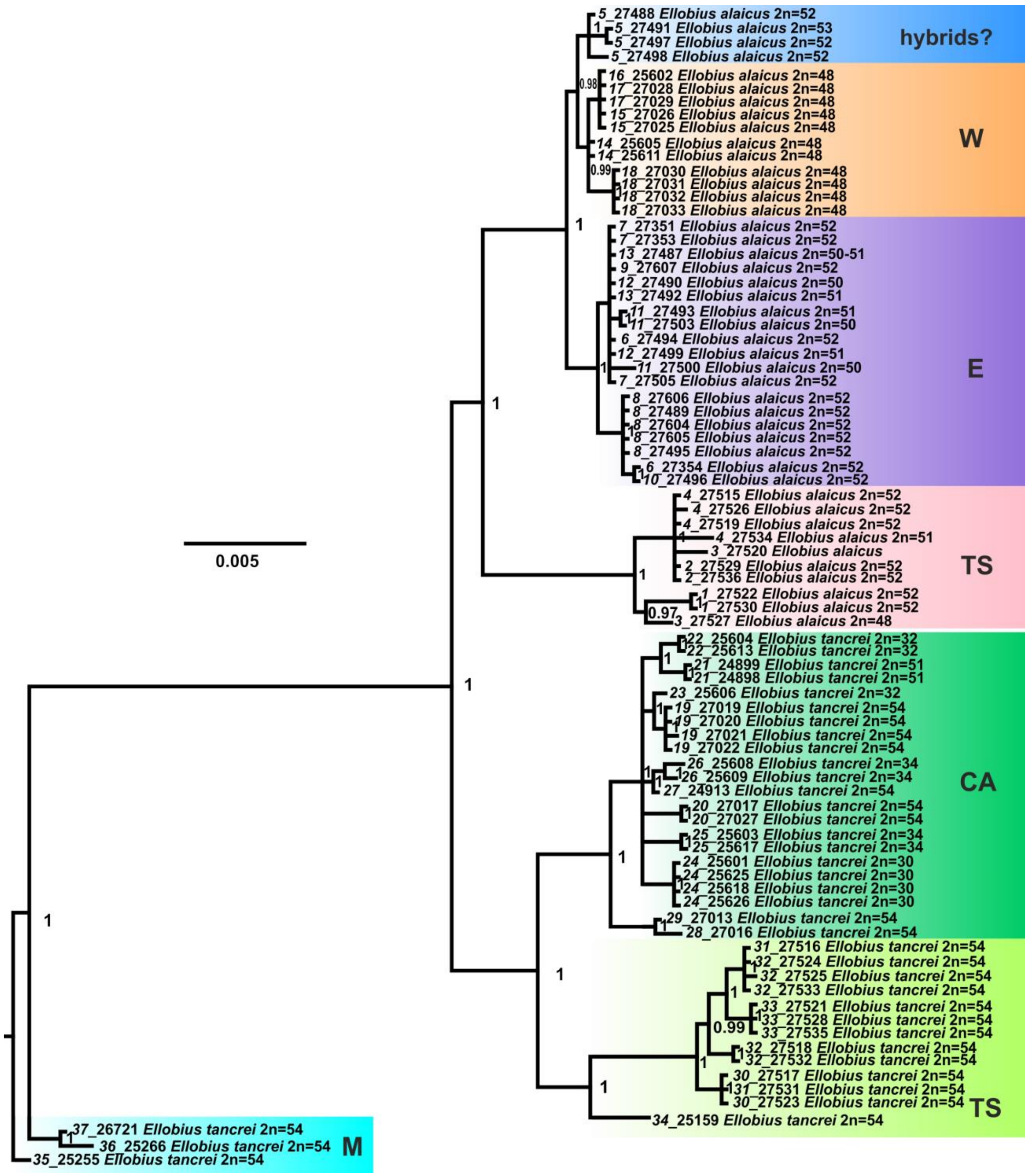

Figure S6. The Bayesian tree based on joined *cytb* gene and *COI* gene sequences of 82 specimens of *E. tancrei* and *E. alaicus*. Samples from *E. tancrei* Mongolian populations were used as an outgroup. Numbers to the right of the nodes correspond to posterior probability values; values <0.70 are not specified. Sample names are presented as "locality number—individual number". The color selection corresponds to haplowebs: cyan marks Mongolian *E. tancrei* (#35–37); deep green marks *E. tancrei* from Tajikistan (#19-29); bright green marks Tien Shan *E. tancrei* (#30-34); blue marks probable interspecific hybrids from Gulcha vicinities (#5); pink marks Tien Shan *E. alaicus* (#1-4); purple and orange mark "E" (#6-10) and "W" (#14-18) haplotypes of *E. alaicus*, respectively. Populations #11-13 belong to "E" group but demonstrated some features (*IRBP* alleles).

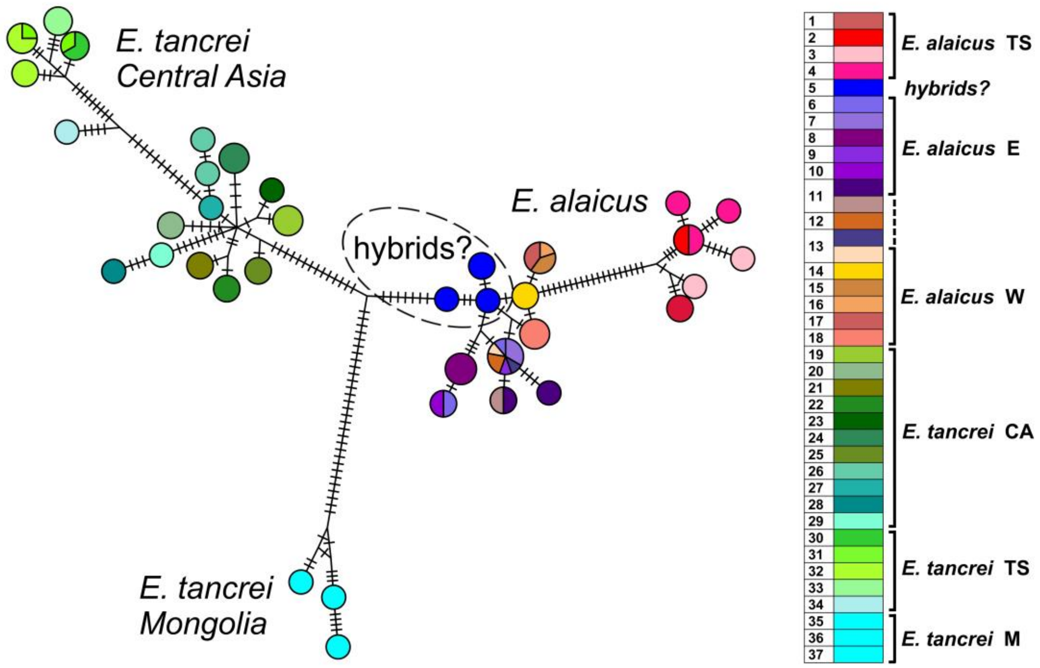

Figure S7. Haplotype network constructed by HaplowebMaker based on *cytb* gene sequences of 82 specimens of *E. tancrei* and *E. alaicus* (see Table S1). A sample from each locality is colored with its own color; close shades unite species/intraspecific forms, according to investigated taxonomic hypotheses. The circles represent haplotypes, the curves between them indicate hybrid individuals, carrying both connected haplotypes. The area of the circles and the thickness of the curves are proportional to the number of individuals carrying the haplotype; nucleotide substitutions are indicated by dashes. Dotted line indicates probable interspecific hybrids (#5).

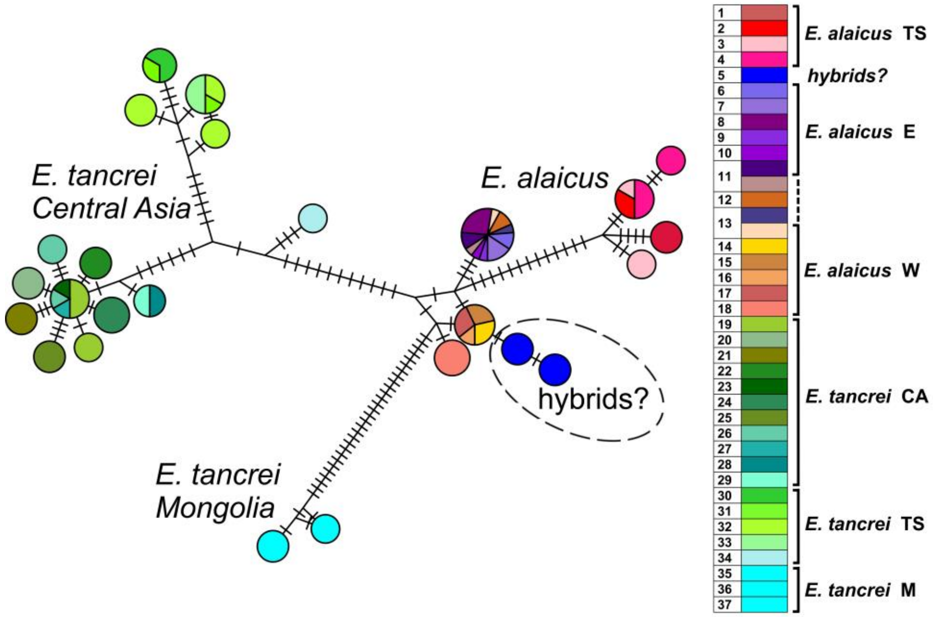

Figure S8. Haplotype network constructed by HaplowebMaker based on *COI* gene sequences of 82 specimens of *E. tancrei* and *E. alaicus* (see Table S1). A sample from each locality is colored with its own color; close shades unite species/intraspecific forms, according to investigated taxonomic hypotheses. The circles represent haplotypes, the curves between them indicate hybrid individuals, carrying both connected haplotypes. The area of the circles and the thickness of the curves are proportional to the number of individuals carrying the haplotype; nucleotide substitutions are indicated by dashes. Dotted line indicates probable interspecific hybrids (#5).

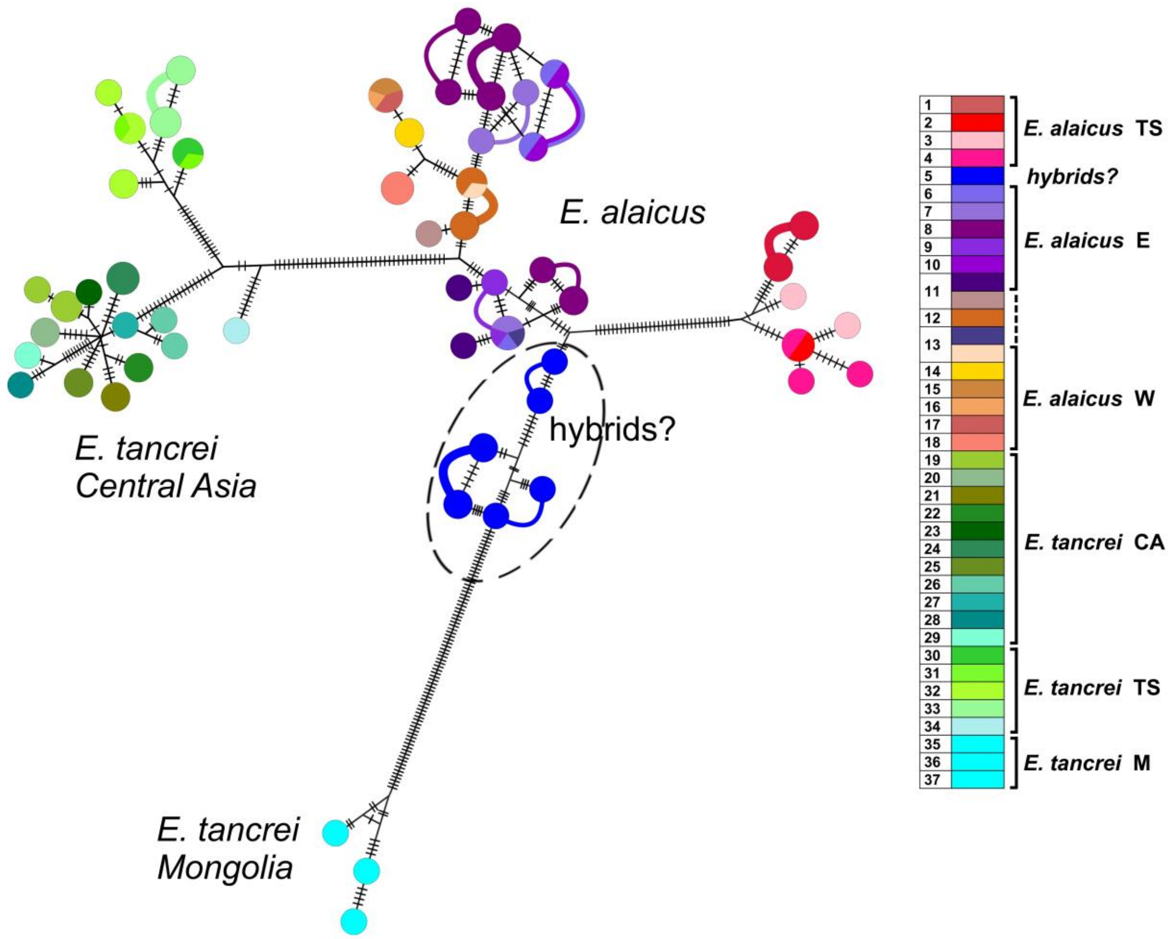

Figure S9. Haplotype network constructed by HaplowebMaker based on the sequences of two mitochondrial (*cytb* and *COI*) and two nuclear (*XIST* and *IRBP*) genes of 82 specimens of *E. tancrei* and *E. alaicus* (see Table S1). A sample from each locality is colored with its own color; close shades unite species/intraspecific forms, according to investigated taxonomic hypotheses. The circles represent haplotypes, the curves between them indicate hybrid individuals, carrying both connected haplotypes. The area of the circles and the thickness of the curves are proportional to the number of individuals carrying the haplotype; nucleotide substitutions are indicated by dashes. Dotted line indicates probable interspecific hybrids (#5).
