# Supplementary material for: Speciation on the Roof of the World: Parallel Fast Evolution of Cryptic Mole Vole Species in the Pamir-Alay—Tien Shan Region
Source: Life (Basel). 2023 Aug 16;13(8):1751. doi: 10.3390/life13081751 (PMC10455883; doi:10.3390/life13081751)
Supplement: Supplementary file 1 [file life-13-01751-s001.zip › Tables S1_S2_S3.pdf]

Table S1. Material, localities and GenBank accession numbers.

Locality No	Species	Voucher	2n	Rb	Sex	Locality	Coordinates	Colors	GenBank accession numbers				
									cytb	*COI*	*IRBP*	*XIST*, first fragment	*XIST*, second fragment
1	*E. alaicus*	**27522**	52	2 Rb (2.11)	m	Kyrgyzstan, to the south of Kazarman	N 41.24369 E 73.95531	Crimson 220,20,60	**OR231548**	**OR232598**	**OR231574**	**OR231600**	**OR231626**
	E. alaicus	**27530**	52	2 Rb (2.11)	m				**OR231549**	**OR232599**	**OR231575**	**OR231601**	**OR231627**
2	*E. alaicus*	**27529**	52	2 Rb (2.11)	m	Kyrgyzstan, Naryn district, Bosogo	N 41.23357 E 76.41762	Red 255,0,0	**OR231550**	**OR232600**	**OR231576**	**OR231602**	**OR231628**
	E. alaicus	**27536**	52	2 Rb (2.11)	m				**OR231551**	**OR232601**	**OR231577**	**OR231603**	**OR231629**
3	*E. alaicus*	**27520**	-	-	m	Kyrgyzstan, Naryn district, Tash-Bashat	N 41.54442 E 76.4397	Pink 255,192,203	**OR231552**	**OR232602**	**OR231578**	**OR231604**	**OR231630**
	E. alaicus	**27527**	48	2 Rb (2.11), 2 Rb (1.3), 1 Rb (6.8), 1 Rb (5.12)	f				**OR231553**	**OR232603**	**OR231579**	**OR231605**	**OR231631**
4	*E. alaicus*	**27515**	52	2 Rb (2.11)	f	Kyrgyzstan, Naryn district, At-Bashy	N 41.3310 E 76.03983	DeepPink 255,20,147	**OR231554**	**OR232604**	**OR231580**	**OR231606**	**OR231632**
	E. alaicus	**27519**	52	2 Rb (2.11)	m				**OR231555**	**OR232605**	**OR231581**	**OR231607**	**OR231633**
	E. alaicus	**27526**	52	2 Rb (2.11)	m				**OR231556**	**OR232606**	**OR231582**	**OR231608**	**OR231634**
	E. alaicus	**27534**	51	2 Rb (2.11), 1 Rb (1.3)	m				**OR231557**	**OR232607**	**OR231583**	**OR231609**	**OR231635**
5	Possibly, backcrosses of *E. tancrei* and *E. alaicus*	27488	52	2 Rb (2.11)	f	Kyrgyzstan, Pamir highway from Osh to Gulcha (between Gulcha and Tashkoro)	N 40.25278 E 73.31554	Blue 0,0,255	ON333901[3]	**OR232608**	ON333848[3]	ON314941[3]	ON314885[3]
	Possibly, backcrosses of *E. tancrei* and *E. alaicus*	27491	53	1 Rb (2.11)	f				ON333902[3]	**OR232609**	ON333849[3]	ON314942[3]	ON314886[3]

	Possibly, backcrosses of *E. tancrei* and *E. alaicus*	27497	52	2 Rb (2.11)	m				ON333903[3]	**OR232610**	ON333850[3]	ON314943[3]	ON314887[3]
	Possibly, backcrosses of *E. tancrei* and *E. alaicus*	27498	52	2 Rb (2.11)	m				ON333904[3]	**OR232611**	ON333851[3]	ON314944[3]	ON314888[3]
6	*E. alaicus*	27354	52	2 Rb (2.11)	f	Kyrgyzstan, the Taldyk pass	N 39.74833<br>E 73.22766	Medium SlateBlue 123,104,238	ON333905[3]	**OR232612**	ON333852[3]	ON314945[3]	ON314889[3]
	E. alaicus	27494	52	2 Rb (2.11)	m				ON333906[3]	**OR232613**	ON333853[3]	ON314946[3]	ON314890[3]
7	*E. alaicus*	27351	52	2 Rb (2.11)	m	Kyrgyzstan, Alay Valley, Sary-Tash vicinities	N 39.72392<br>E 73.24677	Medium Purple 147,112,219	ON333907[3]	**OR232614**	ON333854[3]	ON314947[3]	ON314891[3]
	E. alaicus	27353	52	2 Rb (2.11)	m				ON333908[3]	**OR232615**	ON333855[3]	ON314948[3]	ON314892[3]
	E. alaicus	27505	52	2 Rb (2.11)	m				ON333909[3]	**OR232616**	ON333856[3]	ON314949[3]	ON314893[3]
8	*E. alaicus*	27489	52	2 Rb (2.11)	f	Kyrgyzstan, Alay Valley, Taunmuruk vicinities	N 39.64459<br>E 73.76935	Purple 128,0,128	ON333910[3]	**OR232617**	ON333857[3]	ON314950[3]	ON314894[3]
	E. alaicus	27495	52	2 Rb (2.11)	m				ON333911[3]	**OR232618**	ON333858[3]	ON314951[3]	ON314895[3]
	E. alaicus	**27604**	52	2 Rb (2.11)	m				**OR231558**	**OR232619**	**OR231584**	**OR231610**	**OR231636**
	E. alaicus	**27605**	52	2 Rb (2.11)	m				**OR231559**	**OR232620**	**OR231585**	**OR231611**	**OR231637**
	E. alaicus	**27606**	52	2 Rb (2.11)	f				**OR231560**	**OR232621**	**OR231586**	**OR231612**	**OR231638**
9	*E. alaicus*	**27607**	52	2 Rb (2.11)	m	Kyrgyzstan, Alay Valley, Bardabo vicinities	N 39.639694<br>E 73.240306	BlueViolet 138,43,226	**OR231561**	**OR232622**	**OR231587**	**OR231613**	**OR231639**
10	*E. alaicus*	27496	52	2 Rb (2.11)	m	Kyrgyzstan, Alay Valley, about 3 km to the west from Sary-Tash	N 39.7029<br>E 73.21874	DarkViolet 148,0,211	ON333912[3]	**OR232623**	ON333859[3]	ON314952[3]	ON314896[3]
11	*E. alaicus*	27493	51	2 Rb (2.11)<br>1 Rb (3.10)	m	Kyrgyzstan, Alay Valley, Sary-Mogol vicinities	N 39.619<br>E 72.60924	Indigo 75,0,130	ON333913[3]	**OR232624**	ON333860[3]	ON314953[3]	ON314897[3]
	E. alaicus	27500	50	2 Rb (2.11)<br>2 Rb (3.10)	m				ON333914[3]	**OR232625**	ON333861[3]	ON314954[3]	ON314898[3]

	E. alaicus	27503	50	2 Rb (2.11)<br>2 Rb (3.10)	m			Rosy Brown 188,143,143	ON333915[3]	**OR232626**	ON333862[3]	ON314955[3]	ON314899[3]
12	*E. alaicus*	27490	50	2 Rb (2.11)<br>2 Rb (3.10)	f	Kyrgyzstan, Alay Valley, Daroot-Korgon, point 1 (near bridge across Kyzylsu)	N 39.53976<br>E 72.1712	Chocolate 210,105,30	ON333916[3]	**OR232627**	ON333863[3]	ON314956[3]	ON314900[3]
		27499	51	2 Rb (2.11)<br>1 Rb (3.10)	m				ON333917[3]	**OR232628**	ON333864[3]	ON314957[3]	ON314901[3]
13	*E. alaicus*	27487	50-51	2 Rb (2.11)<br>1-2 Rb (3.10)	m	Kyrgyzstan, Alay Valley, Daroot-Korgon, point 2	N 39.53918<br>E 72.17201	Dark SlateBlue 72,61,139	ON333918[3]	**OR232629**	ON333865[3]	ON314958[3]	ON314902[3]
	E. alaicus	27492	51	2 Rb (2.11)<br>1 Rb (3.10)	m			PeachPuff 255,218,185	ON333919[3]	**OR232630**	ON333866[3]	ON314959[3]	ON314903[3]
14	*E. alaicus*	25605	48	2 Rb (2.11), 2 Rb (4.9), 2 Rb (3.10)	f	Tajikistan, Pamir-Alay, Achek-Alma vicinities	N 39.37883<br>E 71.678	Gold 255,215,0	MG264322[1]	**OR232631**	ON333867[3]	MK544925[1]	ON314904[3]
	E. alaicus	25611	48	2 Rb (2.11), 2 Rb (4.9), 2 Rb (3.10)	m				MG264324[1]	**OR232632**	ON333868[3]	ON314960[3]	ON314905[3]
15	*E. alaicus*	27025	48	2 Rb (2.11), 2 Rb (4.9), 2 Rb (3.10)	m	Tajikistan, Pamir-Alay, Dzhailgan vicinities	N 39.32128<br>E 71.54537	Peru 205,133,63	MK544910[1]	**OR232633**	ON333869[3]	MK544926[1]	ON314906[3]
	E. alaicus	27026	48	2 Rb (2.11), 2 Rb (4.9), 2 Rb (3.10)	m				MK544911[1]	**OR232634**	ON333870[3]	ON314961[3]	ON314907[3]
16	*E. alaicus*	25602	48	2 Rb (2.11), 2 Rb (4.9), 2 Rb (3.10)	f	Tajikistan, Pamir-Alay, Duvana vicinities	N 39.345<br>E 71.57883	Sandy Brown 244,164,96	MG264326[1]	**OR232635**	ON333871[3]	MK544924[1]	ON314908[3]
17	*E. alaicus*	27028	48	2 Rb (2.11), 2 Rb (4.9), 2 Rb (3.10)	f	Tajikistan, Pamir-Alay, Kashat vicinities, near bridge across Kyzylsu	N 39.30748<br>E 71.47467	IndianRed 205,92,92	MK544913[1]	**OR232636**	ON333872[3]	ON314962[3]	ON314909[3]

	E. alaicus	27029	48	2 Rb (2.11), 2 Rb (4.9), 2 Rb (3.10)	f				MK544914[1]	**OR232637**	ON333873[3]	ON314963[3]	ON314910[3]
18	*E. alaicus*	27030	48	2 Rb (2.11), 2 Rb (4.9), 2 Rb (3.10)	f	Tajikistan, Pamir-Alay, Muksu River, left bank	N 39.2458 E 71.41667	Salmon 250,128,114	MK544915[1]	**OR232638**	ON333874[3]	ON314964[3]	ON314911[3]
	E. alaicus	27031	48	2 Rb (2.11), 2 Rb (4.9), 2 Rb (3.10)	f				ON333920[3]	**OR232639**	ON333875[3]	ON314965[3]	ON314912[3]
	E. alaicus	27032	48	2 Rb (2.11), 2 Rb (4.9), 2 Rb (3.10)	f				MK544916[1]	**OR232640**	ON333876[3]	ON314966[3]	ON314913[3]
	E. alaicus	27033	48	2 Rb (2.11), 2 Rb (4.9), 2 Rb (3.10)	m				MK544917[1]	**OR232641**	ON333877[3]	ON314967[3]	ON314914[3]
19	*E. tancrei*	27019	54	no Rbs	m	Tajikistan, Pamir-Alay, Utol-Poyon vicinities	N 39.16228 E 71.1229	Yellow Green 154,205,50	MK544906[1]	**OR232642**	MT478770[2]	ON314968[3]	ON314915[3]
	E. tancrei	27020	54	no Rbs	f				MK544907[1]	**OR232643**	ON333878[3]	ON314969[3]	ON314916[3]
	E. tancrei	27021	54	no Rbs	m				MK544908[1]	**OR232644**	ON333879[3]	ON314970[3]	ON314917[3]
	E. tancrei	27022	54	no Rbs	f				MK544909[1]	**OR232645**	ON333880[3]	ON314971[3]	ON314918[3]
20	*E. tancrei*	27017	54	no Rbs	m	Tajikistan, Pamir-Alay, between Kichikzy and Utol-Poyon	N 39.12708 E 70.99603	DarkSea Green 143,188,143	MK544904[1]	**OR232646**	ON333881[3]	MK544923[1]	ON314919[3]
	E. tancrei	27027	54	no Rbs	m				MK544912[1]	**OR232647**	ON333882[3]	ON314972[3]	ON314920[3]
21	*E. tancrei*	24898	52	see [3]	m	Tajikistan, Pamir-Alay, Kichikzy vicinities	N 39.823 E 70.5733	Olive 128,128,0	ON333921[3]	**OR232648**	ON333883[3]	ON314973[3]	ON314921[3]
	E. tancrei	24899	51	see [3]	f				ON333922[3]	**OR232649**	ON333884[3]	ON314974[3]	ON314922[3]

22	*E. tancrei*	25604	32	see [3]	m	Tajikistan, Pamir-Alay, Sarinai vicinities	N 39.0645 E 70.8716	Forest Green 34,139,34	ON333923[3]	**OR232650**	ON333885[3]	ON314975[3]	ON314923[3]
	E. tancrei	25613	32	see [3]	m				ON333924[3]	**OR232651**	ON333886[3]	ON314976[3]	ON314924[3]
23	*E. tancrei*	25606	32	see [3]	m	Tajikistan, Pamir-Alay, Obi-Kaboud	N 39.22527 E 70.85218	DarkGreen 0,100,0	ON333925[3]	**OR232652**	ON333887[3]	ON314977[3]	ON314925[3]
24	*E. tancrei*	25601	30	see [4]	f	Tajikistan, Pamir-Alay, Shilbili vicinities	N 39.25617 E 71.34317	SeaGreen 46,139,87	MG264327[1]	**OR232653**	ON333888[3]	ON314978[3]	ON314926[3]
	E. tancrei	25618	30	see [4]	f				MG264328[1]	**OR232654**	ON333889[3]	ON314979[3]	ON314927[3]
	E. tancrei	25625	30	see [4]	m				MG264329[1]	**OR232655**	ON333890[3]	ON314980[3]	ON314928[3]
	E. tancrei	25626	30	see [4]	f				MG264330[1]	**OR232656**	ON333891[3]	ON314981[3]	ON314929[3]
25	*E. tancrei*	25603	34	see [3]	m	Tajikistan, Pamir-Alay, Khozar-Chashma vicinities	N 39.03083 E 70.50472	OliveDrab 107,142,35	ON333926[3]	**OR232657**	ON333892[3]	ON314982[3]	ON314930[3]
	E. tancrei	25617	34	see [3]	m				ON333927[3]	**OR232658**	ON333893[3]	ON314983[3]	ON314931[3]
26	*E. tancrei*	25608	34	see [3]	m	Tajikistan, Pamir-Alay, Saripoul vicinities	N 38.8775 E 70.0007	Medium Aquama-rine 102,205,170	ON333928[3]	**OR232659**	ON333894[3]	ON314984[3]	ON314932[3]
	E. tancrei	25609	34	see [3]	f				ON333929[3]	**OR232660**	ON333895[3]	ON314985[3]	ON314933[3]
27	*E. tancrei*	24913	54	no Rbs	m	Tajikistan, Pamir-Alay, Romit	N 38.75455 E 69.29332	LightSea Green 32,178,170	MG264345[1]	**OR232661**	MT478769[2]	MK544921[1]	ON314934[3]
28	*E. tancrei*	27016	54	no Rbs	m	Tajikistan, Sovetabad vicinities	N 37.47465 E 68.25947	DarkCyan 0,139,139	MK544903[1]	**OR232662**	ON333896[3]	ON314986[3]	ON314935[3]
29	*E. tancrei*	27013	54	no Rbs	m	Tajikistan, Aivadj vicinities	N 36.96947 E 68.01152	Aquama-rine 127,255,212	MK544901[1]	**OR232663**	ON333897[3]	ON314987[3]	ON314936[3]
30	*E. tancrei*	**27517**	54	no Rbs	m	Kyrgyzstan, Song Kol lake, point 1	N 41.941158 E 75.091455	LimeGreen 50,205,50	**OR231562**	**OR232664**	**OR231588**	**OR231614**	**OR231640**
	E. tancrei	**27523**	54	no Rbs	m				**OR231563**	**OR232665**	**OR231589**	**OR231615**	**OR231641**
31	*E. tancrei*	**27516**	54	no Rbs	f	Kyrgyzstan, Song Kol lake, point 2	N 41.87815 E 75.30855	Lawn Green 124,252,0	**OR231564**	**OR232666**	**OR231590**	**OR231616**	**OR231642**
	E. tancrei	**27531**	54	no Rbs	m				**OR231565**	**OR232667**	**OR231591**	**OR231617**	**OR231643**

32	*E. tancrei*	**27518**	54	no Rbs	m	Kyrgyzstan, Sary-Bulak vicinities	N 41.922600 E 75.741380	Green Yellow 173,255,47	**OR231566**	**OR232668**	**OR231592**	**OR231618**	**OR231644**
	E. tancrei	**27524**	54	no Rbs	m				**OR231567**	**OR232669**	**OR231593**	**OR231619**	**OR231645**
	E. tancrei	**27525**	54	no Rbs	m				**OR231568**	**OR232670**	**OR231594**	**OR231620**	**OR231646**
	E. tancrei	**27532**	54	no Rbs	m				**OR231569**	**OR232671**	**OR231595**	**OR231621**	**OR231647**
	E. tancrei	**27533**	54	no Rbs	m				**OR231570**	**OR232672**	**OR231596**	**OR231622**	**OR231648**
33	*E. tancrei*	**27521**	54	no Rbs	m	Kyrgyzstan, between Sary-Bulak and Naryn	N 41.697870 E 75.787990	PaleGreen 152,251,152	**OR231571**	**OR232673**	**OR231597**	**OR231623**	**OR231649**
	E. tancrei	**27528**	54	no Rbs	m				**OR231572**	**OR232674**	**OR231598**	**OR231624**	**OR231650**
	E. tancrei	**27535**	54	no Rbs	m				**OR231573**	**OR232675**	**OR231599**	**OR231625**	**OR231651**
34	*E. tancrei*	25159	54	no Rbs	m	Uzbekistan, Tashkent	N 41.3415 E 70.31183	PaleTur-quoise 175,238,238	MG264346[1]	**OR232676**	MT478768[2]	MK544922[1]	ON314937[3]
35	*E. tancrei*	25255	54	no Rbs	-	Mongolia, Bulgan	N 46.95417 E 91.19139	Cyan 0,255,255	MT468380[2]	**OR232677**	ON333898[3]	ON314988[3]	ON314938[3]
36	*E. tancrei*	25266	54	no Rbs	f	Mongolia, Khargantyn Ula	N 49.15428 E 89.92281		ON333930[3]	**OR232678**	ON333899[3]	ON314989[3]	ON314939[3]
37	*E. tancrei*	26721	54	no Rbs	f	Mongolia, Arakhangai	N 47.347500 E 101.844444		ON333931[3]	**OR232679**	ON333900[3]	ON314990[3]	ON314940[3]

Notes: [1] – cited in [11], [2] – cited in [8], [3] – cited in [12].
Voucher numbers and GenBank numbers of individuals, which were firstly analyzed in the present study, are highlighted in bold.
Populations #11-13 belong to "E" group of *E. alaicus* but demonstrated some features (*IRBP* alleles), so they were marked by several color shades.

Table S2. The mean *p*-distance values calculated between different intraspecific groups of *E. alaicus* and *E. tancrei*, as well between these species in total, on the basis of analysis of the *cytb* gene (the upper line in each table cell), the *COI* gene fragment (the intermediate line), and joined sequences of them (the bottom line). Intraspecific groups of *E. alaicus* and *E. tancrei* correspond to those given in Figure S2.

Species and intraspecific forms	*p*-distances										
	E. alaicus (TS)	*E. alaicus* (E)	*E. alaicus* (W)	Hybrids from #5 Gulcha	***E. alaicus* from Pamir-Alay: E+W+ hybrids**	***E. alaicus* in total**	*E. tancrei* (T)	*E. tancrei* (TS, exclu-ding #34 Tashkent)	*E. tancrei* (#34 Tashkent)	***E. tancrei* (TS clade in total)**	**Central Asian *E. tancrei*: T+TS total clade**
E. alaicus (E)	0.023<br>0.017<br>0.020	—									
E. alaicus (W)	0.021<br>0.016<br>0.018	0.004<br>0.004<br>0.004	—								
Hybrids from #5 Gulcha	0.022<br>0.017<br>0.019	0.004<br>0.005<br>0.004	0.002<br>0.002<br>0.002	—							
***E. alaicus* from Pamir-Alay: E+W+hybrids**	**0.022**<br>**0.017**<br>**0.020**	—	—	—	—						
E. tancrei (T)	0.030<br>0.021<br>0.026	0.024<br>0.020<br>0.022	0.025<br>0.018<br>0.022	0.023<br>0.020<br>0.021	**0.024**<br>**0.020**<br>**0.022**	**0.026**<br>**0.020**<br>**0.023**	—				
E. tancrei (TS, excluding #34 Tashkent)	0.031<br>0.025<br>0.028	0.028<br>0.022<br>0.025	0.027<br>0.019<br>0.023	0.026<br>0.020<br>0.023	**0.027**<br>**0.021**<br>**0.024**	**0.028**<br>**0.022**<br>**0.025**	0.022<br>0.015<br>0.019	—			
E. tancrei (#34 Tashkent)	0.032<br>0.019<br>0.026	0.025<br>0.017<br>0.021	0.025<br>0.015<br>0.020	0.024<br>0.014<br>0.019	**0.025**<br>**0.016**<br>**0.020**	**0.026**<br>**0.017**<br>**0.022**	0.017<br>0.011<br>0.014	0.013<br>0.011<br>0.012	—		
***E. tancrei* (TS clade in total)**	**0.031**<br>**0.024**<br>**0.028**	**0.027**<br>**0.022**<br>**0.025**	**0.027**<br>**0.019**<br>**0.023**	**0.025**<br>**0.020**<br>**0.023**	**0.027**<br>**0.021**<br>**0.024**	**0.028**<br>**0.021**<br>**0.025**	**0.022**<br>**0.014**<br>**0.018**	—	—	—	
**Central Asian *E. tancrei*: T+TS total clade**	**0.030**<br>**0.022**<br>**0.026**	**0.025**<br>**0.021**<br>**0.023**	**0.025**<br>**0.018**<br>**0.022**	**0.024**<br>**0.020**<br>**0.022**	**0.025**<br>**0.020**<br>**0.023**	**0.026**<br>**0.020**<br>**0.023**	—	—	—	—	—
E. tancrei (M)	0.048<br>0.036<br>0.042	0.040<br>0.031<br>0.036	0.039<br>0.029<br>0.034	0.037<br>0.031<br>0.034	**0.039**<br>**0.031**<br>**0.035**	**0.041**<br>**0.032**<br>**0.037**	0.042<br>0.037<br>0.040	0.047<br>0.038<br>0.042	0.048<br>0.034<br>0.041	**0.047**<br>**0.037**<br>**0.042**	**0.044**<br>**0.037**<br>**0.041**

Table S3. The mean *p*-distance values calculated within different intraspecific groups of *E. alaicus* and *E. tancrei*, as well within these species in total, on the basis of analysis of the *cytb* gene (the upper line in each table cell), the *COI* gene fragment (the intermediate line), and joined sequences of them (the bottom line). Intraspecific groups of *E. alaicus* and *E. tancrei* correspond to those given in the Figure S2.

Species and intraspecific forms	*p*-distances
E. alaicus<br>(TS)	0.004<br>0.003<br>0.003
E. alaicus<br>(E)	0.002<br>0.000<br>0.001
E. alaicus<br>(W)	0.001<br>0.001<br>0.001
Hybrids from #5 Gulcha	0.001<br>0.001<br>0.001
***E. alaicus* from Pamir-Alay:**<br>**E+W+hybrids**	**0.003**<br>**0.003**<br>**0.003**
***E. alaicus* in total**	**0.010**<br>**0.008**<br>**0.009**
E. tancrei<br>(T)	0.005<br>0.002<br>0.004
E. tancrei<br>(TS, excluding #34 Tashkent)	0.003<br>0.002<br>0.003
E. tancrei<br>(#34 Tashkent)	—
E. tancrei<br>**(TS clade in total)**	**0.005**<br>**0.003**<br>**0.004**
**Central Asian**<br>***E. tancrei*:**<br>**T+TS total clade**	**0.013**<br>**0.008**<br>**0.011**
E. tancrei<br>(M)	0.006<br>0.001<br>0.004
